# Genome-Wide Identification and Expression Profiling of *ABA-Stress-Ripening* (*ASR*) Gene Family in Barley (*Hordeum vulgare* L.)

**DOI:** 10.3390/plants14060970

**Published:** 2025-03-19

**Authors:** Jie Ren, Kangfeng Cai, Xiujuan Song, Wenhao Yue, Lei Liu, Fangying Ge, Qiuyu Wang, Junmei Wang

**Affiliations:** 1 Institute of Crop and Nuclear Technology Utilization, Zhejiang Academy of Agricultural Sciences, Hangzhou 310021, China; renjiexzy@163.com (J.R.); caikf@zaas.ac.cn (K.C.); song05@stu.zafu.edu.cn (X.S.); yuewh@zaas.ac.cn (W.Y.); liulei453@126.com (L.L.); gefy@stu.zafu.edu.cn (F.G.); wangqy@zjnu.edu.cn (Q.W.); 2 Agricultural Technology Extension Center, Deqing Bureau of Agriculture and Rural Affairs, Deqing 313200, China; 3 National Barley Improvement Centre, Zhejiang Academy of Agricultural Sciences, Hangzhou 310021, China; 4 College of Life Sciences, Zhejiang Normal University, Jinhua 321004, China

**Keywords:** ASR, barley, expression, abiotic stress response, transcription factor

## Abstract

Abscisic acid (ABA)-stress-ripening, or ABA-, stress-, and ripening-induced (ASR) proteins play an important role in responses to environmental stimuli. A total of ten barley *HvASRs* were identified in this study, which were unevenly distributed on three chromosomes. ASRs from barley, wheat, *Brachypodium distachyon*, rice, maize, foxtail millet, and tomato were classified into two distinct clusters based on phylogenetic analysis. Notably, ASRs from Poaceae were evenly distributed between these two clusters. HvASRs contained a typical ABA/WDS domain, and exhibited similar motif arrangements. Two gene pairs of tandem duplicates (*HvASR4/5/6/7* and *HvASR8/9*) were identified among *HvASRs*. *Cis*-acting elements involved in hormone and stress responses, including ABRE, MYB, ARE, and STRE, were consistently identified in the promoters of *HvASRs*. The expression of *HvASRs* was substantially influenced by salt, osmotic, and ABA treatments in the roots and leaves of barley seedlings. *HvASR2* acts as a transcriptional repressor, whereas *HvASR3* serves as a transcriptional activator. These results enhance our understanding of the *HvASR* family and provide a foundation for further functional characterization.

## 1. Introduction

*Abscisic acid* (*ABA*)*-stress-ripening*, or *ABA-*, *stress-*, and *ripening-induced* (*ASR*) genes encode plant-specific, low-molecular-weight, and highly hydrophilic proteins possessing conserved abscisic acid/water deficit stress (ABA/WDS) domains. ABA/WDS is intrinsically disordered, allowing its conformation to adapt in support of the diverse functions of ASRs [1]. Since the initial identification of an *ASR* gene from a tomato fruit cDNA library in 1993 [2], more and more *ASRs* have been identified among gymnosperms, monocots, and dicots [3]. However, *ASRs* are notably absent in Brassicaceae species, including the model plant Arabidopsis.

ASRs exhibit a dual nature, functioning as either transcription factors or chaperones. The unstructured form of tomato SlASR1 has been reported to display chaperone-like activity in the cytosol, effectively preventing protein denaturation during repeated freeze–thaw cycles [4]. Similar results have also been observed in ASRs from plantain [5] and lily [6]. On the other hand, SlASR1 exhibits zinc-dependent DNA-binding activity, and the overexpression of *SlASR1* significantly enhances salt tolerance in transgenic tobacco plants [7]. BdASR1 from *Brachypodium distachyon* acts as a transcription factor and confers drought tolerance by enhancing antioxidant capacity [8].

ASRs play crucial roles in responses to external stimuli. *OsASR1* is induced by low temperature, and overexpression of *OsASR1* improves photosynthetic capacity and cold stress tolerance in transgenic rice plants [9]. OsASR1 also has been verified to be a positive regulator of the ABA signaling pathway and enhance the drought and salinity stress tolerance of transgenic plants by improving osmolyte accumulation, stomatal closure modulation, and transpiration rates [10]. OsASR5 acts as a chaperone-like protein and improves drought tolerance via regulating the ABA pathway and promoting stomatal closure [11]. OsASR6 serves predominantly as a functional protein and enhances salt tolerance through the modulation of reactive oxygen species (ROS) homeostasis and ABA signaling [12]. The overexpression of *SiASR1* significantly enhances tolerance to drought and oxidative stresses in tobacco plants [13]. *SiASR4*, as the target gene of SiARDP, enhances drought and salt tolerance in transgenic Arabidopsis and foxtail millet plants through ROS- and ABA-dependent pathways [14]. Constitutive overexpression of *TaASR1-D* improves tolerance to multiple stresses, including oxidative, osmotic, drought, and salinity stress, by enhancing the antioxidant system and ABA signaling [15]. Heterologous expression of *TaASR2D* in *Brachypodium distachyon* increases the expression of stress-related and ABA-responsive genes, and endogenous levels of ABA and hydrogen peroxide, and increases stomatal closure under drought stress, thereby leading to improved drought tolerance [16]. ZmASR3 positively regulates drought tolerance by maintaining water content, increasing ABA content and stomatal closure, reducing ROS accumulation, and inducing the expression of genes involved in ROS-related, stress-responsive, and ABA-dependent pathways [17]. Overexpression of *BdASR4* leads to enhanced drought tolerance by activating the antioxidant defense system and inducing the expression of genes involved in stress- and ABA-response pathways [18]. *SlASR1* [19], *SbASR-1* [20,21], *PheASR2* [22], *ThASR3* [23], and *HaASR2* [24] have also been demonstrated to enhance abiotic stresses in transgenic plants. In addition, *ASR* genes were also found to function in cadmium tolerance [25] and aluminum tolerance [26].

The *ASR* family has been identified in many species such as rice [27], maize [25,28], wheat [15,29,30], *Brachypodium distachyon* [8], foxtail millet [13,31], and tomato [32,33]. However, such identification has not been conducted in barley. This work identified a total of 10 *HvASRs* in barley, and subsequently conducted comprehensive analyses. The response patterns of *HvASRs* to salt, osmotic, and ABA treatments in barley seedling roots and leaves were examined, and their transcriptional activation capacity was investigated using a Y2HGold yeast system. The results will establish a solid foundation for the functional characterization of *HvASRs*.

## 2. Results

### 2.1. Barley HvASRs Identification

In total, 10 barley *HvASR* genes were identified (Table 1). The amino acid lengths of *HvASRs* varied from 97 amino acids (aas) to 282 aas. Their isoelectric point (pI) and molecular weight (MW) were 5.21–9.99 kDa and 10.65–29.95 kDa, respectively (Table 1). The instability index varied from 28.89 (HvASR11) to 50.62 (HvASR4). The instability indices of HvASR3/5/6/8/9/10 were <40, suggesting that these proteins are relatively stable [34]. The aliphatic indices and grand average of hydropathicity (GRAVY) of HvASRs were 18.05–67.24 and −1.68–−0.78, respectively, indicating that HvASRs were all hydrophilic proteins. Disordered amino acids of HvASRs varied from 59.57% to 89.34%. HvASRs were predicted to localize in the nucleus, except HvASR2, which was localized in the chloroplast (Table 1). *HvASR10* was previously designated as *HvASR1* in an earlier study [35]. In a separate investigation, *HvASR10* was functionally characterized and named *HvASR5* for its similarity to *OsASR5* in rice [36].

### 2.2. Phylogenetic Relationships and Gene Structures of HvASRs

**Figure 1:**
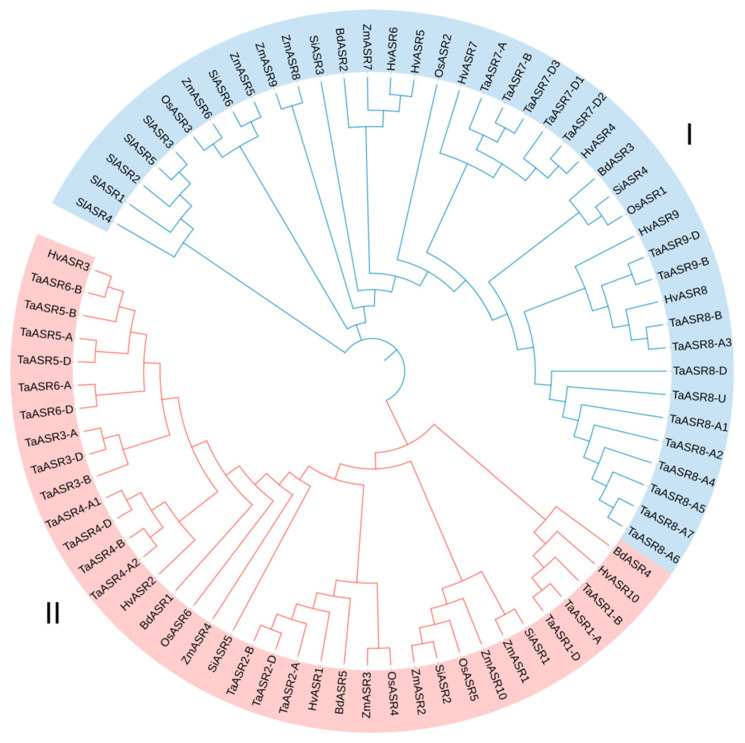
Phylogenetic tree of *ASR* family proteins from rice, maize, wheat, *Brachypodium distachyon*, foxtail millet, tomato, and barley. Amino acid sequences of all ASRs were aligned with MAFFT, and a phylogenetic tree was constructed using MEGA X with the maximum-likelihood method and 1000 bootstraps. Os: *Oryza sativa*; Zm: *Zea mays*; Ta: *Triticum aestivum*; Bd: *Brachypodium distachyon*; Si: *Setaria italica*; Sl: *Solanum lycopersicum*; Hv: *Hordeum vulgare*.

The amino acid sequences of 6 *OsASRs* in rice, 10 *ZmASRs* in maize, 36 *TaASRs* in wheat, 5 *BdASRs* in *Brachypodium distachyon*, 6 *SiASRs* in foxtail millet, 5 *SlASRs* in tomato, and 10 *HvASRs* were used for phylogenetic analysis (Figure 1). These 78 ASRs could be classified into two clusters, Cluster I and Cluster II. Cluster I contained 41 members, and Cluster II contained 37 members. SlASRs were all in Cluster I, whereas 60% of HvASRs (6) were in Cluster I. The ASRs of the remaining species were distributed almost evenly between Cluster I and Cluster II.

**Figure 2:**
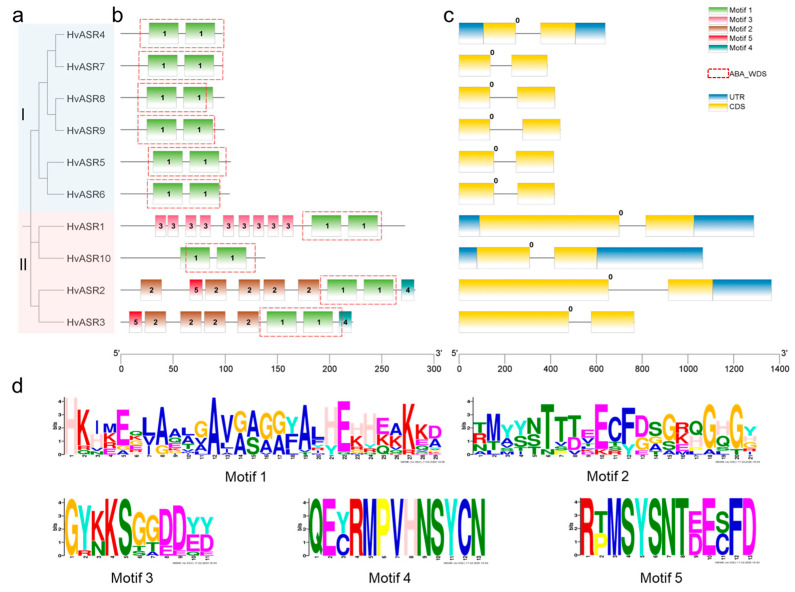
Phylogeny and sequence characteristics of *HvASRs*. (**a**) Phylogenetic tree of *HvASRs*, constructed using MEGA X with maximum-likelihood method and 1000 bootstraps. (**b**) Conserved motifs of *HvASRs*. (**c**) Gene structures of *HvASRs*. (**d**) Amino acid sequences of motifs; the uppercase letters designate one-letter abbreviation of amino acids.

All HvASRs possessed an ABA/WDS domain, and HvASRs from the identical cluster exhibited comparable motif configurations (Figure 2a,b). The amino acid sequences of motif 1 to motif 5 were “HKIKEKLAALGAVAAGGYALHEHHEAKKD”, “TMYYNTTTEECFDSGKQGHGY”, “GYKKSGGDDED”, “QECRMPVHNSYCN”, and “RPMSYSNTEECFD”, respectively (Figure 2d). The ABA/WDS domain consisted of two of motif 1; therefore, all HvASRs contained two motif 1 elements. In contrast, motif 3, motif 4, and motif 5 exhibited gene-specific distribution and were present only in specific HvASR members. Introns are classified into three kinds based on the positional relationship relative to codons, namely phase 0, phase 1, and phase 2. Phase 0 introns are situated between two codons, whereas phase 1 introns and phase 2 introns are located after the first and second positions within a codon, respectively [37]. Intriguingly, all *HvASRs* contained one phase 0 intron (Figure 2c).

### 2.3. HvASRs Distribution and Duplication

**Figure 3:**
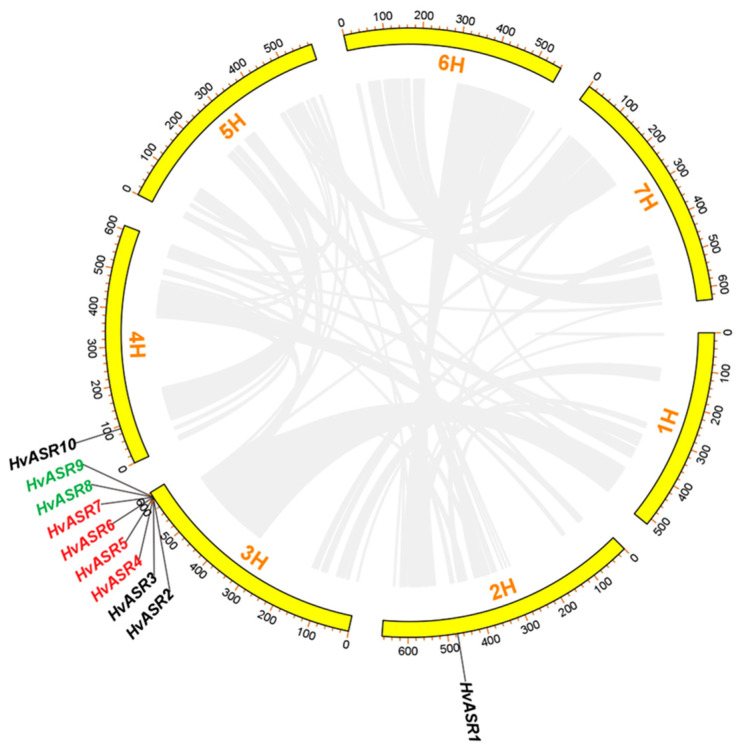
Chromosomal distribution and tandem duplication of *HvASRs*. Duplication events were analyzed using Tbtools v2.210. Two tandemly duplicated gene pairs are displayed in red and green, respectively.

Ten *HvASRs* were unevenly distributed across three out of seven chromosomes, with eight genes on chromosome 3, and one gene each on chromosome 2 and 4, respectively (Figure 3). Gene duplication serves as a primary mechanism driving gene family expansion [38]. Tandem duplicated genes refer to adjacent homologous genes located on the same chromosome, with no more than one intervening gene [39]. Two gene pairs of tandem duplicates (*HvASR4/5/6/7* and *HvASR8/9*) were identified among *HvASRs*. However, no segmental duplication events were detected.

### 2.4. Cis-Acting Elements

**Figure 4:**
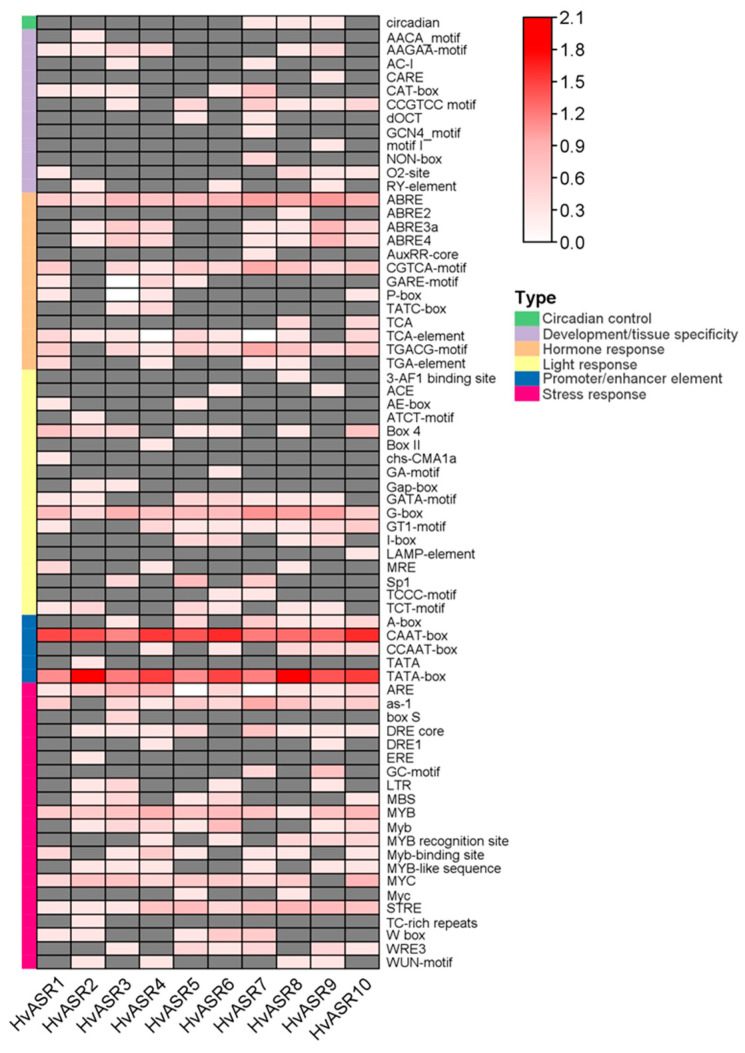
*Cis*-acting elements in the promoters of *HvASRs*. A total of 2 kb of upstream regions of all *HvASRs* were used for analysis through PlantCARE. The absent elements are displayed in a grey color.

Seventy *cis*-acting elements were detected in *HvASR* promoters. These elements were classified into six classes (Figure 4; File S1). The “Stress response” category contained the most elements (21, 30.0%), followed by “light response” (18, 25.7%), “hormone response” (13, 18.6%), “development/tissue specificity” (12, 17.1%), and “promoter/enhancer element” (5, 7.1%). The “Circadian control” category had only one element. A TATA box and CAAT box were present in all *HvASR* promoters, suggesting their pivotal roles in transcription regulation. In the “hormone response” category, the ABA responsiveness-related element, ABRE, was widely distributed in *HvASR* promoters. G-box in the “light response” was also prevalent in the promoters of *HvASRs* (Figure 4). Furthermore, elements in “stress response”, such as ARE, MYB, and STRE, were also ubiquitously present in the promoters of all *HvASRs*. However, other elements were comparatively gene-specific. Thus, *HvASRs* may play crucial roles in external stimuli responses.

### 2.5. Syntenic Relationships of ASRs Between Barley and Other Species

**Figure 5:**
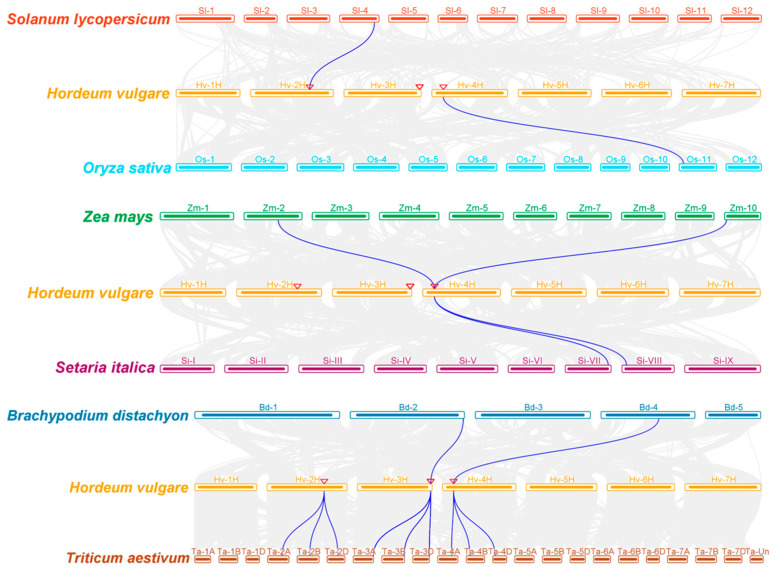
Syntenic relationships of *ASRs* between barley and six plant species (*Solanum lycopersicum*, *Oryza sativa*, *Zea mays*, *Setaria italica*, Brachypodium distachyon, and *Triticum aestivum*). Syntenic relationships were analyzed using TBtools. Syntenic blocks are indicated with grey lines, and syntenic *ASR* gene pairs are indicated with blue lines. Triangles indicate the positions of *HvASRs*.

The synteny of *ASRs* between barley and other plant species were investigated (Figure 5; File S2). There was only one pair of orthologs each, observed between barley and tomato and between barley and rice, namely *HvASR1*/*SlASR3* and *HvASR10*/*OsASR5*, respectively. A comparison of barley to maize, foxtail millet, and *Brachypodium distachyon* showed the presence of two pairs of orthologous genes, namely *HvASR10*/*ZmASR1* and *HvASR10*/*ZmASR2*, and *HvASR10*/*SiASR1* and *HvASR10*/*SiASR2*, as well as *HvASR2*/*BdASR1* and *HvASR10*/*BdASR4*, respectively. Wheat is more closely related to barley than the other five plant species; thus, the comparison between these two species led to substantially more orthologous gene pairs (15 pairs). Each of *HvASR1*/*2*/*8*/*10* was orthologous to three *TaASRs*, and each of *HvASR4*/*5*/*7* was orthologous to one *TaASR* gene. *HvASR3*, *HvASR6*, and *HvASR9* had no orthologs within the wheat genome.

### 2.6. Tissue-Specific Expression of HvASRs

**Figure 6:**
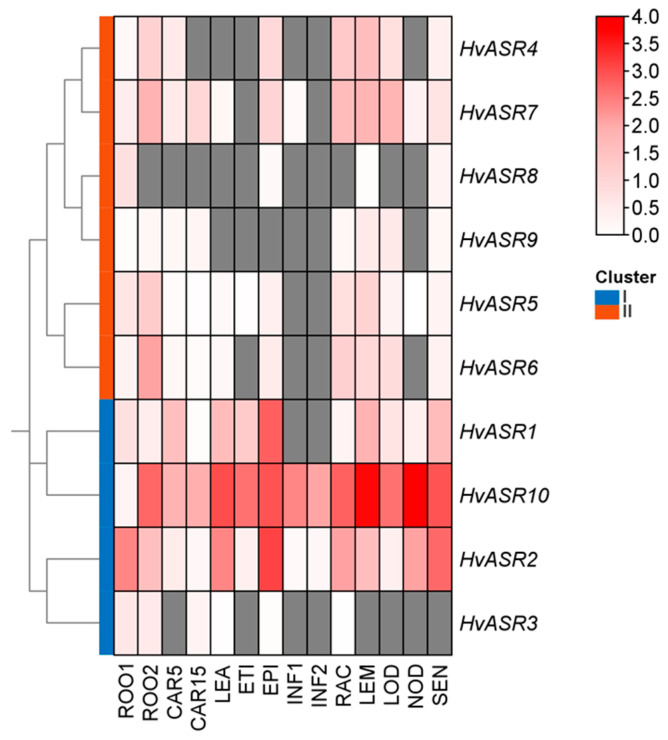
Expression levels of *HvASRs* in 14 tissues. Transcriptomic data were downloaded from BARLEX, and FPKM values were normalized using log_10_(FPKM+1) transformation. The heatmap was drawn using TBtools. ROO1, roots from seedlings (10 cm shoot stage); ROO2, roots (28 DAP); CAR5, developing grain (5 DAP); CAR15, developing grain (15 DAP); LEA, shoots from seedlings (10 cm shoot stage); ETI, etiolated seedling, dark condition (10 DAP); EPI, epidermal strips (28 DAP); INF1, young developing inflorescences (5 mm); INF2, developing inflorescences (1–1.5 cm); RAC, inflorescences, rachis (35 DAP); LEM, inflorescences, lemma (42 DAP); LOD, inflorescences, lodicule (42 DAP); NOD, developing tillers, third internode (42 DAP); SEN, senescing leaves (56 DAP). Grey squares indicate that gene expression was not detected.

The expression levels of *HvASRs* displayed dramatic variation among 14 different tissues, and *HvASRs* in Cluster I exhibited comparably higher expression levels than those in Cluster II (Figure 6; File S3). The expression of *HvASR2* and *HvASR10* within Cluster I was detected across all tissue types and developmental stages, whereas the expression of the other *HvASRs* was relatively tissue-specific and of low abundance. In addition, the expression of *HvASR8* was detected in roots from seedlings at the 10 cm shoot stage, whereas expression was absent in roots at 28 days after pollination, suggesting the growth stage also matters.

### 2.7. Responses of HvASRs to Salt and Osmotic Stress and Exogenous ABA Treatment

**Figure 7:**
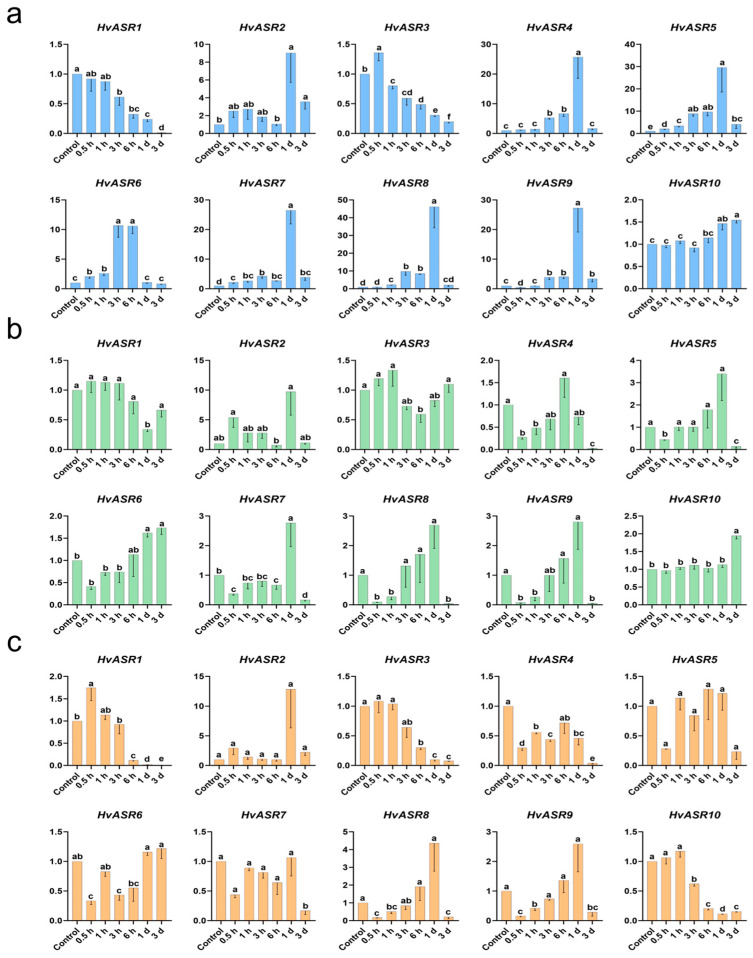
Expression responses of *HvASRs* in barley seedling roots after salt stress (**a**), osmotic stress (**b**), and ABA treatments (**c**). Relative expression levels of *HvASRs* were calculated using the 2^−ΔΔCT^ method. Lowercase letters indicate significance analysis performed using a threshold of *p* < 0.05.

**Figure 8:**
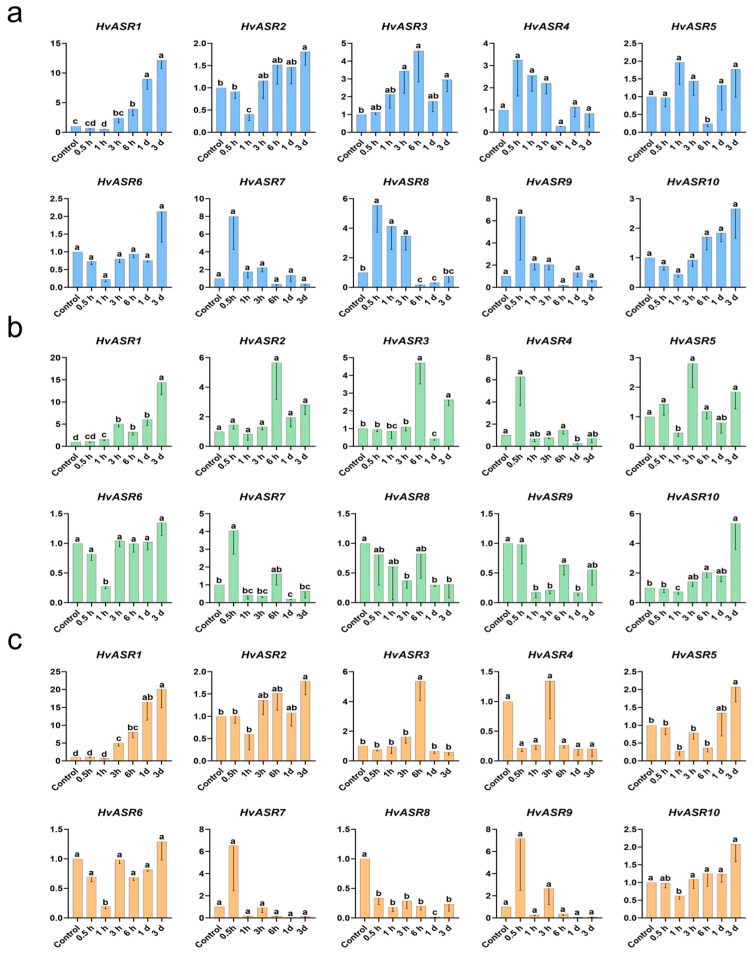
Expression responses of *HvASRs* in barley seedling leaves after salt stress (**a**), osmotic stress (**b**), and ABA treatments (**c**). Relative expression levels of *HvASRs* were calculated using the 2^−ΔΔCT^ method. Lowercase letters indicate significance analysis performed using a threshold of *p* < 0.05.

The expression of *HvASRs* in response to salt and osmotic stresses, as well as exogenous ABA treatment, were further examined in the roots and leaves of barley seedlings (Figure 7 and Figure 8). *HvASRs* were significantly induced or inhibited in response to stress treatments, albeit to varying degrees. In roots, the expression of *HvASR1* was progressively inhibited by salt stress, with the degree of inhibition intensifying as the treatment duration extended (Figure 7a). *HvASR2* was slightly induced by salt stress at 0.5 h, and thereafter progressively inhibited as treatment duration extended. *HvASR2*/*4*/*5*/*7*/*8*/*9* were dramatically induced (9.0-fold to 46.2-fold of control), and their expression levels peaked after salt treatment for 1 d. *HvASR6* was also induced by salt stress, but its expression level peaked at 3 h and 6 h. However, the expression of *HvASR10* was relatively stable. The response of *HvASRs* to osmotic stress and ABA treatment were considerably less pronounced in comparison to salt stress (Figure 7a,b). *HvASR2* was the gene that was induced the most; the expression level of which reached 9.7-fold of control after osmotic stress treatment for 1 d. Interestingly, the expression of *HvASR4/5/7*/*8*/*9* was significantly decreased upon the initiation of osmotic stress, then increased and peaked after treatment for 1 d, and subsequently dramatically decreased after treatment for 3 d. The expression of *HvASR1/3* alternated between induction and suppression, and that of *HvASR10* was significantly induced only after osmotic stress for 3 d. The expression pattern of *HvASR2* in response to ABA treatment was analogous to its response to osmotic stress, with the highest induction and peak expression occurring after a 1 day of ABA treatment (Figure 7c). The response patterns of *HvASR8*/*9* after ABA treatment resembled that under osmotic stress conditions (Figure 7b,c). *HvASR4* was consistently suppressed during the whole period of ABA treatment, whereas *HvASR1*/*3*/*10* were significantly inhibited at the late stage of treatment (6 h to 3 d).

The expression levels of *HvASR1* and *HvASR3* in leaves were induced by salt stress, exhibiting opposite response patterns compared with those in roots (Figure 7a and Figure 8a). The expression of the other *HvASRs* was dynamic, alternating between induction and suppression (Figure 8a). The expression of *HvASR1* was continuously upregulated, whereas that of *HvASR8* and *HvASR9* was steadily downregulated in response to osmotic stress (Figure 8b). The expression levels of the other *HvASRs* fluctuated over time. The expression of *HvASR1* was induced, whereas that of *HvASR8* was continuously suppressed after exogenous ABA treatment (Figure 8c). The expression of *HvASR2/3/4/5/6/7/9/10* was relatively dynamic.

### 2.8. Transcriptional Activation Capacity of HvASRs

**Figure 9:**
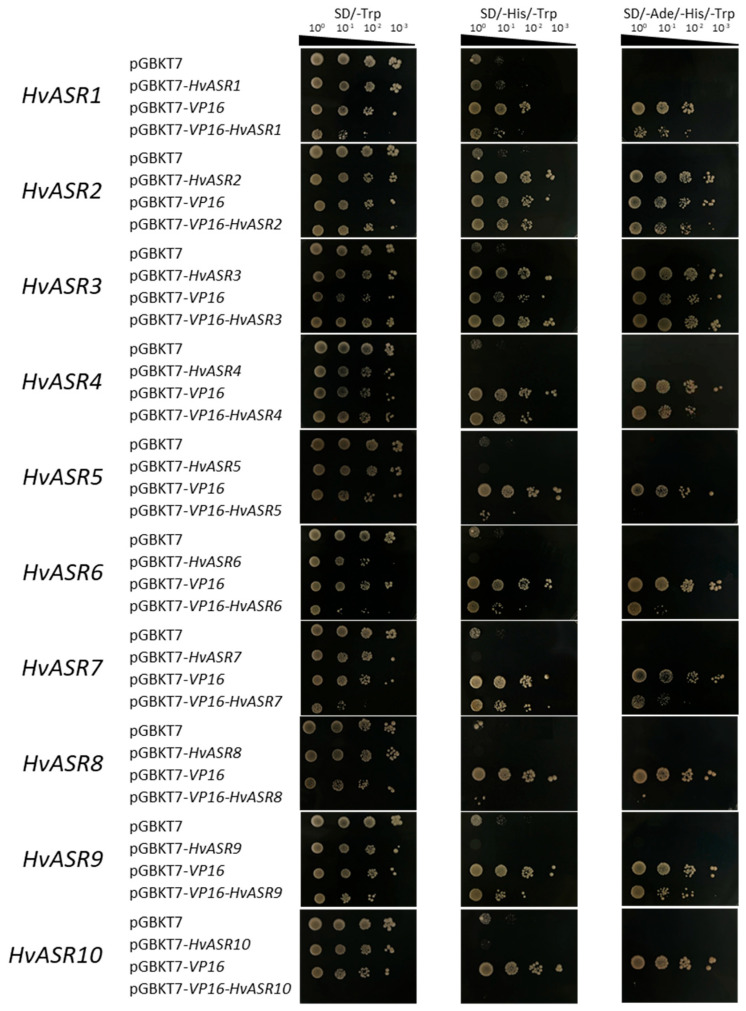
Transcriptional activity analyses of HvASR proteins. *HvASRs* were cloned and inserted into pGBKT7 and pGBKT7-*VP16* vectors. Subsequently, pGBKT7, pGBKT7-*VP16*, and the recombinant vectors were transformed into Y2HGold yeast strains, respectively, and cultured on SD/-Trp, SD/-His/-Trp, and SD/-Ade/-His/-Trp plates for 3 d.

ASRs function as either chaperones or transcription factors. The transcriptional activation capacity of *HvASRs* was analyzed using the Y2HGold yeast system (Figure 9). Y2HGold yeast strains expressing pGBKT7 exhibited limited growth on SD/-His/-Trp plates, and were unable to grow on SD/-Ade/-His/-Trp plates. Herpes simplex virus protein VP16 can activate the transcription of target genes, and GAL4-VP16 is a transcriptional activator, which is formed by fusion of the yeast activator GAL4 to VP16 [40]. GAL4-VP16 is capable of activating the expression of *HIS3* and *ADE2*, thereby rescuing the growth of Y2HGold yeast strains on SD/-Ade/-His/-Trp plates. Yeast strains expressing *HvASR2* and *HvASR3* succeeded in growing on SD/-Ade/-His/-Trp plates, demonstrating that these two genes possess transcriptional activation capacity. The expression of *HvASR2* inhibited the growth of yeast strains expressing pGBKT7-*VP16*, while the expression of *HvASR3* enhanced such growth on SD/-Ade/-His/-Trp plates. This suggested that *HvASR2* acts as a transcriptional repressor, whereas *HvASR3* functions as a transcriptional activator. Notably, the yeast strains expressing pGBKT7-*VP16*-*HvASR5*, pGBKT7-*VP16*-*HvASR8*, and pGBKT7-*VP16*-*HvASR10* failed to grow on SD/-Trp plates. The authors speculated that the fusion expression of *HvASR5*, *HvASR8*, and *HvASR10* might have interfered with the function of GAL4-VP16 through an unknown interaction.

## 3. Discussion

ASRs were classified into two clusters according to phylogenetic relationships (Figure 1). All SlASRs from tomato were in Cluster I, whereas ASRs from other species including barley, wheat, rice, maize, *Brachypodium distachyon*, and foxtail millet were almost distributed evenly between Cluster I and Cluster II. The protein lengths of ASRs from Cluster II were substantially longer than those from Cluster I (File S4). In terms of ASRs in barley, HvASR1/2/3/10 were classified into Cluster II with an average length of 228 amino acids, which was 2.28 times the average length of the other HvASRs in Cluster I (Figure 1; File S4). Notably, as the evolutionary distance to barley increased, the length ratio of Cluster II to Cluster I progressively decreased, declining from 2.02 in wheat to 1.34 in maize (File S4).

*ZmASR7*, *ZmASR8*, and *ZmASR9* in maize were devoid of introns, whereas the other *ZmASRs* had one intron [28]. Two *TaASRs* in wheat had two introns, and the remaining *TaASRs* contained one intron [29,30]. *MsASR6* was intron-rich with four introns, whereas the others in *Miscanthus* had no or only one intron [41]. All *HvASRs* in barley contained one intron (Figure 2c), which was also observed among BdASRs in *Brachypodium distachyon* [8] and *TtASRs* in *Tetragonia tetragonoides* [42]. Introns may impede prompt regulatory responses and are, therefore, subject to negative selection in genes that necessitate rapid regulation during stress responses, which represents an internal mechanism driving the extensive loss of introns observed in certain eukaryotic lineages throughout evolution. [43]. Based on this perspective, *HvASRs* might be fast-responding genes upon environmental stimuli.

A total of 23 pairs of tandem duplicated genes and six pairs of segmentally duplicated genes were identified within 29 wheat *TaASRs* [29]. On the other hand, 33 *TaASRs* were identified in other research, and 14 pairs of *TaASRs* were tandem duplicated, and eight *TaASR* groups were segmentally duplicated [30]. Two pairs of *TtASRs* were segmentally duplicated in *Tetragonia tetragonoides* [42]. However, among the *HvASRs* in barley, only two gene pairs of tandem duplicates (*HvASR4*/*5*/*6*/*7* and *HvASR8*/*9*) were detected, while no segmental duplication events were observed (Figure 3). These results indicated that plant species took different strategies for *ASR* gene family expansion during evolution.

The expression of *HvASR2* and *HvASR10* was detected across all tissues and stages, whereas that of the other *HvASRs* was comparatively low and tissue-specific (Figure 6; File S3). Furthermore, *HvASR10* exhibited a much higher expression level compared to the other *HvASR* genes across most tissues and developmental stages. Tissue-specific expression of *ASRs* was also observed in tomato [33], wheat [30], maize [28], and foxtail millet [31]. *Cis*-acting elements, such as ABRE in the “hormone response” category, G-box in the “light response” category, and ARE, MYB, and STRE in the “stress response” category, were ubiquitous in the promoters of *HvASRs* (Figure 4). Similar phenomena were also observed in *MsASRs* from *Miscanthus* [41]. The widespread occurrence of *cis*-acting elements associated with hormone signaling and external stimuli suggested a significant role for *HvASRs* in responding to environmental cues. The responses of *HvASRs* to salt, osmotic, and ABA treatments in the roots and leaves of barley seedlings were further examined via qRT-PCR (Figure 7 and Figure 8). The expression of *HvASRs* was significantly induced or inhibited by salt, osmotic, and ABA treatments, albeit to varying degrees (Figure 7 and Figure 8). Several *HvASRs* displayed opposite response patterns in roots and leaves, such as *HvASR1* and *HvASR3* under salt stress conditions (Figure 7a and Figure 8a). After 1 d of salt, osmotic, and ABA treatments, HvASR2 was significantly upregulated in roots, whereas it was only slightly induced in leaves without reaching statistical significance (Figure 7 and Figure 8), indicating that tissue type also affected expression patterns. The responses of *HvASRs* to osmotic stress and ABA treatment were considerably less pronounced in comparison to salt stress (Figure 7 and Figure 8), which might be explained by the complex nature of salt stress comprising osmotic stress, ionic stress, and oxidative stress [44].

Some ASRs were proved to be intrinsically disordered proteins, such as TtASR1 in wheat [35], SbASR-1 in *Salicornia brachiata* [21], PgASR3 in pearl millet [45], and SlASR in *Suaeda liaotungensis* [46]. HvASR10 was composed of 84.78% disordered amino acids, and was also proved to be an intrinsically disordered protein in previous research (designated as HvASR1 therein) [35]. HvASR2 and HvASR3 were composed of 59.57% and 67.57% disordered amino acids, respectively, whereas the other HvASRs consisted of >70% disordered amino acids (Table 1). A transcriptional activation assay revealed that the expression of *HvASR2* and *HvASR3* restored yeast growth on SD/-Ade/-His/-Trp plates, indicating the transcriptional activation capacity of *HvASR2* and *HvASR3* (Figure 9). Thus, *HvASR2* and *HvASR3* may function as transcription factors, whereas the remaining *HvASRs* function as molecular chaperones. HvASR2 and HvASR3 were both in Cluster II and were closer to each other than any other HvASRs based on phylogenetic analysis (Figure 2a). In addition, motif 2, motif 4, and motif 5 were exclusively present in HvASR2 and HvASR3 (Figure 2b), which might lead to their transcriptional activation capacity. Similarly, twelve *TaASRs* were proved to possess activation activity in yeast cells [29], and *BdASR1* was also evidenced to be a transcription factor [8]. In addition, the expression of *HvASR2* enhanced the growth of yeast strains expressing pGBKT7-*VP16*, while the expression of *HvASR3* inhibited such growth on SD/-Ade/-His/-Trp plates (Figure 9), indicating that *HvASR2* functions as a transcriptional repressor, whereas *HvASR3* serves as a transcriptional activator.

## 4. Materials and Methods

### 4.1. Identification of HvASRs

The protein sequences of *ASRs* from tomato [32] and rice [27] were used as a query for a blast search in barley. Meanwhile, “IPR003496” and “PF02496” were used to search *ASR* candidates in the barley genome in Ensembl Plants (http://plants.ensembl.org/, accessed on 16 October 2024). The putative *HvASRs* were further validated in InterPro [47]. In the end, 10 *HvASR* genes were identified.

### 4.2. Properties and Localizations of HvASRs

The pI, MW, instability indices, aliphatic indices, and GRAVY of HvASRs were predicted using PROTPARAM [48]. Disordered amino acids and subcellular localizations were analyzed using PrDOS [49] and BUSCA [50], respectively.

### 4.3. Phylogeny, Synteny, and Duplication Analyses

ASR amino acid sequences from barley (10), rice (6), maize (10), *Brachypodium distachyon* (5), wheat (36), foxtail millet (6), and tomato (5) were aligned using MAFFT [51], and phylogenetic trees were built using MEGA X 1.0 (maximum-likelihood method with 1000 bootstraps) [52]. Syntenic relationships and duplication events were analyzed using TBtools [53,54].

### 4.4. Sequence, Promoter, and Expression Analyses

Conserved motifs were analyzed on MEME Suite 5.5.2 (classic mode with five motifs and any number of repetitions) [55,56]. *Cis*-acting elements within the 2 kb upstream regions of *HvASRs* were identified through PlantCARE [57]. Transcriptomic data (FPKM) were downloaded from BARLEX (Accession: PRJEB14349) [58,59], and visualized using TBtools v2.210 [53].

### 4.5. Plant Growth, Treatments, and qRT-PCR

Barley (cv. Morex) seeds were germinated and grown in a 1/5 Hoagland solution for 10 d. Then, salt stress (200 mM NaCl), osmotic stress (20% PEG8000), and ABA treatments (100 μM ABA) were imposed on the seedlings [60]. Seedlings grown in normal nutrient solution were set as a control. After treatments for 0.5 h, 1 h, 3 h, 6 h, 1 d, and 3 d, roots were sampled in three replicates for qRT-PCR [60]. A MiniBEST Plant RNA Extraction Kit (9769, TaKaRa, Shiga, Japan) and PrimeScript RT Master Mix (RR036A, TaKaRa, Shiga, Japan) were used for RNA extraction and cDNA synthesis, respectively. ChamQ Universal SYBR qPCR Master Mix (Q711, Vazyme, Nanjing, China) was used for qRT-PCR with an LC480 II (Roche, Basel, Switzerland), with two technical replicates [61]. Relative expression levels of *HvASRs* were calculated using the 2^−ΔΔCT^ method [62], with actin as the reference gene [54]. The primers used are listed in File S5.

### 4.6. Transcriptional Activation Capacity Assay

First-strand cDNA synthesis and amplification were performed as described in [61]. The gene-specific primers are listed in File S6. *HvASRs* were cloned into pYES2-NTB plasmids using EcoRI (R0101V, New England Biolabs, Ipswich, MA, USA) and ClonExpress II One Step Cloning Kit (C112, Vazyme, Nanjing, China). *HvASR2* and *HvASR8* exhibited high similarity in the 3′ end of the coding sequence to *HvASR3* and *HvASR9*, respectively; thus, they were de novo synthesized into pYES2-NTB plasmids (SUNYA, Hangzhou, China). Subsequently, *HvASRs* were reconstructed into pGBKT7 and pGBKT7-*VP16* plasmids, respectively (File S7). Recombinant vectors were transformed into Y2HGold yeast cells, and the transcriptional activity assay was conducted following instructions (MH102, Coolaber, Beijing, China).

## 5. Conclusions

A total of ten barley *HvASRs* were identified. All HvASRs comprised a typical domain of ABA/WDS and displayed conserved motif configurations. Two gene pairs of tandem duplicates (*HvASR4/5/6/7* and *HvASR8/9*) were identified among *HvASRs*. Hormone- and stress response-related *cis*-acting elements, including ABRE, ARE, MYB, and STRE, were ubiquitous in *HvASR* promoters. The expression of *HvASRs* was significantly affected by salt, osmotic, and exogenous ABA treatments, with varying expression levels observed in roots and leaves. *HvASR2* might be a transcriptional repressor, whereas *HvASR3* serves as a transcriptional activator, and both warrant further investigation.

## Figures and Tables

**Table 1:** Characteristics of 10 *HvASR* genes in barley.

Gene Name	Gene ID	Length (aas)	pI	MW (kDa)	Instability Index	Aliphatic Index	GRAVY	Disordered aas (%)	Subcellular Localization
*HvASR1*	HORVU.MOREX.r3.2HG0168120	272	5.21	29.59	41.95	18.05	−1.68	89.34	nucleus
*HvASR2*	HORVU.MOREX.r3.3HG0325150	282	6.59	29.95	50.62	35.07	−0.89	59.57	chloroplast
*HvASR3*	HORVU.MOREX.r3.3HG0325170	222	6.40	23.86	38.19	29.14	−1.00	67.57	nucleus
*HvASR4*	HORVU.MOREX.r3.3HG0325200	97	9.82	10.76	42.80	48.45	−1.22	73.20	nucleus
*HvASR5*	HORVU.MOREX.r3.3HG0325210	105	9.52	11.11	36.32	67.24	−0.78	76.19	nucleus
*HvASR6*	HORVU.MOREX.r3.3HG0325230	104	9.40	11.06	35.54	65.10	−0.84	75.00	nucleus
*HvASR7*	HORVU.MOREX.r3.3HG0325240	97	9.87	10.65	42.05	49.59	−1.13	71.13	nucleus
*HvASR8*	HORVU.MOREX.r3.3HG0325300	99	9.99	11.04	34.67	49.60	−1.34	73.74	nucleus
*HvASR9*	HORVU.MOREX.r3.3HG0325310	99	9.94	11.08	38.93	46.67	−1.29	74.75	nucleus
*HvASR10*	HORVU.MOREX.r3.4HG0349450	138	6.14	15.43	28.89	43.33	−1.20	84.78	nucleus

aas: amino acids; pI: isoelectric point; MW: molecular weight; GRAVY: grand average of hydropathicity.

## Data Availability

Data is contained within the article and Supplementary Materials.

## Supplementary Materials

The following supporting information can be downloaded at https://www.mdpi.com/article/10.3390/plants14060970/s1: File S1. *Cis*-acting elements in the promoters of *HvASRs*; File S2. Orthologous *ASR* gene pairs in synteny analysis; File S3. Tissue expression patterns of *HvASRs*; File S4. Amino acid length of HvASRs from different clusters and evolutionary timescale of barley, wheat, *Brachypodium distachyon*, rice, foxtail millet, and maize; File S5. Primers for qRT-PCR; File S6. Primers for cloning of *HvASRs*; File S7. Primers for transcriptional activity assay of *HvASRs*.

## References

[1] Yacoubi I., Hamdi K., Fourquet P., Bignon C., Longhi S. (2021). Structural and Functional Characterization of the ABA-Water Deficit Stress Domain from Wheat and Barley: An Intrinsically Disordered Domain Behind the Versatile Functions of the Plant Abscissic Acid, Stress and Ripening Protein Family. Int. J. Mol. Sci..

[2] Iusem N.D., Bartholomew D.M., Hitz W.D., Scolnik P.A. (1993). Tomato (*Lycopersicon esculentum*) Transcript Induced by Water Deficit and Ripening. Plant Physiol..

[3] González R.M., Iusem N.D. (2014). Twenty Years of Research on *Asr* (ABA-Stress-Ripening) Genes and Proteins. Planta.

[4] Konrad Z., Bar-Zvi D. (2008). Synergism Between the Chaperone-like Activity of the Stress Regulated ASR1 Protein and the Osmolyte Glycine-Betaine. Planta.

[5] Dai J.-R., Liu B., Feng D.-R., Liu H., He Y., Qi K., Wang H.-B., Wang J.-F. (2011). *MpAsr* Encodes an Intrinsically Unstructured Protein and Enhances Osmotic Tolerance in Transgenic *Arabidopsis*. Plant Cell Rep..

[6] Hsu Y.-F., Yu S.-C., Yang C.-Y., Wang C.-S. (2011). Lily ASR Protein-Conferred Cold and Freezing Resistance in Arabidopsis. Plant Physiol. Biochem. PPB.

[7] Goldgur Y., Rom S., Ghirlando R., Shkolnik D., Shadrin N., Konrad Z., Bar-Zvi D. (2006). Desiccation and Zinc Binding Induce Transition of Tomato Abscisic Acid Stress Ripening 1, a Water Stress- and Salt Stress-Regulated Plant-Specific Protein, from Unfolded to Folded State. Plant Physiol..

[8] Wang L., Hu W., Feng J., Yang X., Huang Q., Xiao J., Liu Y., Yang G., He G. (2016). Identification of the *ASR* Gene Family from *Brachypodium distachyon* and Functional Characterization of *BdASR1* in Response to Drought Stress. Plant Cell Rep..

[9] Kim S.-J., Lee S.-C., Hong S.K., An K., An G., Kim S.-R. (2009). Ectopic Expression of a Cold-Responsive *OsAsr1* cDNA Gives Enhanced Cold Tolerance in Transgenic Rice Plants. Mol. Cells.

[10] Park S.I., Kim J.J., Shin S.Y., Kim Y.S., Yoon H.S. (2020). *ASR* Enhances Environmental Stress Tolerance and Improves Grain Yield by Modulating Stomatal Closure in Rice. Front. Plant Sci..

[11] Li J., Li Y., Yin Z., Jiang J., Zhang M., Guo X., Ye Z., Zhao Y., Xiong H., Zhang Z. (2017). *OsASR5* Enhances Drought Tolerance through a Stomatal Closure Pathway Associated with ABA and H_2_O_2_ Signalling in Rice. Plant Biotechnol. J..

[12] Zhang Q., Liu Y., Jiang Y., Li A., Cheng B., Wu J. (2022). *OsASR6* Enhances Salt Stress Tolerance in Rice. Int. J. Mol. Sci..

[13] Feng Z.J., Xu Z.S., Sun J., Li L.C., Chen M., Yang G.X., He G.Y., Ma Y.Z. (2016). Investigation of the ASR Family in Foxtail Millet and the Role of ASR1 in Drought/Oxidative Stress Tolerance. Plant Cell Rep..

[14] Li J., Dong Y., Li C., Pan Y., Yu J. (2017). *SiASR4*, the Target Gene of SiARDP from *Setaria italica*, Improves Abiotic Stress Adaption in Plants. Front. Plant Sci..

[15] Qiu D., Hu W., Zhou Y., Xiao J., Hu R., Wei Q., Zhang Y., Feng J., Sun F., Sun J. (2021). TaASR1-D Confers Abiotic Stress Resistance by Affecting ROS Accumulation and ABA Signalling in Transgenic Wheat. Plant Biotechnol. J..

[16] Yoon J.S., Kim J.Y., Kim D.Y., Seo Y.W. (2021). A Novel Wheat *ASR* Gene, *TaASR2D*, Enhances Drought Tolerance in *Brachypodium distachyon*. Plant Physiol. Biochem..

[17] Liang Y., Jiang Y., Du M., Li B., Chen L., Chen M., Jin D., Wu J. (2019). *ZmASR3* from the Maize *ASR* Gene Family Positively Regulates Drought Tolerance in Transgenic Arabidopsis. Int. J. Mol. Sci..

[18] Yoon J.S., Kim J.Y., Lee M.B., Seo Y.W. (2019). Over-Expression of the *Brachypodium ASR* Gene, *BdASR4*, Enhances Drought Tolerance in *Brachypodium distachyon*. Plant Cell Rep..

[19] Kalifa Y., Perlson E., Gilad A., Konrad Z., Scolnik P.A., Bar-Zvi D. (2004). Over-Expression of the Water and Salt Stress-Regulated *Asr1* Gene Confers an Increased Salt Tolerance. Plant Cell Environ..

[20] Tiwari V., Chaturvedi A.K., Mishra A., Jha B. (2015). Introgression of the *SbASR-1* Gene Cloned from a Halophyte *Salicornia brachiata* Enhances Salinity and Drought Endurance in Transgenic Groundnut (*Arachis hypogaea*) and Acts as a Transcription Factor. PLoS ONE.

[21] Jha B., Lal S., Tiwari V., Yadav S.K., Agarwal P.K. (2012). The *SbASR-1* Gene Cloned from an Extreme Halophyte *Salicornia brachiata* Enhances Salt Tolerance in Transgenic Tobacco. Mar. Biotechnol..

[22] Wu M., Liu R., Gao Y., Xiong R., Shi Y., Xiang Y. (2020). *PheASR2*, a Novel Stress-Responsive Transcription Factor from Moso Bamboo (*Phyllostachys edulis*), Enhances Drought Tolerance in Transgenic Rice via Increased Sensitivity to Abscisic Acid. Plant Physiol. Biochem..

[23] Zhang Y., Ma H., Zhou T., Zhu Z., Zhang Y., Zhao X., Wang C. (2022). *ThASR3* Confers Salt and Osmotic Stress Tolerances in Transgenic *Tamarix* and *Arabidopsis*. BMC Plant Biol..

[24] Cao Y.H., Ren W., Gao H.J., Lü X.P., Zhao Q., Zhang H., Rensing C., Zhang J.L. (2023). *HaASR2* from *Haloxylon ammodendron* Confers Drought and Salt Tolerance in Plants. Plant Sci..

[25] Zhang J., Zhu Q., Yu H., Li L., Zhang G., Chen X., Jiang M., Tan M. (2019). Comprehensive Analysis of the Cadmium Tolerance of Abscisic Acid-, Stress-and Ripening-Induced Proteins (ASRs) in Maize. Int. J. Mol. Sci..

[26] Arenhart R.A., Schunemann M., Bucker Neto L., Margis R., Wang Z.Y., Margis-Pinheiro M. (2016). Rice *ASR1* and *ASR5* Are Complementary Transcription Factors Regulating Aluminium Responsive Genes. Plant Cell Environ..

[27] Pérez-Díaz J., Wu T.M., Pérez-Díaz R., Ruíz-Lara S., Hong C.Y., Casaretto J.A. (2014). Organ- and Stress-Specific Expression of the *ASR* Genes in Rice. Plant Cell Rep..

[28] Zhang L., Yi X., Guo J., Zhao H., Xia Q., He P., Huo S., Qu J., Cao Y., Tan Y. (2021). Expression Patterns of the *ZmASR* Family During Simulated Drought Stress in Maize. Agron. J..

[29] Zan T., Li L., Xie T., Zhang L., Li X. (2020). Genome-Wide Identification and Abiotic Stress Response Patterns of Abscisic Acid Stress Ripening Protein Family Members in *Triticum aestivum* L. Genomics.

[30] Li H., Guan H., Zhuo Q., Wang Z., Li S., Si J., Zhang B., Feng B., Kong L.A., Wang F. (2020). Genome-Wide Characterization of the *Abscisic Acid-*, *Stress-* and *Ripening-Induced (ASR)* Gene Family in Wheat (*Triticum aestivum* L.). Biol. Res..

[31] Cao X.N., Shen L.H., Song J., Wang J.J., Wang H.G., Chen L., Pei Y.X., Liu S.C., Qiao Z.J. (2021). Analysis of Cloned Sequences and Expression of *Asr* Gene Family in Millet. J. Anim. Plant Sci..

[32] Jia H., Jiu S., Zhang C., Wang C., Tariq P., Liu Z., Wang B., Cui L., Fang J. (2016). Abscisic Acid and Sucrose Regulate Tomato and Strawberry Fruit Ripening through the Abscisic Acid-Stress-Ripening Transcription Factor. Plant Biotechnol. J..

[33] Golan I., Guadalupe Dominguez P., Konrad Z., Shkolnik-Inbar D., Carrari F., Bar-Zvi D. (2014). Tomato *ABSCISIC ACID STRESS RIPENING (ASR)* Gene Family Revisited. PLoS ONE.

[34] Guruprasad K., Reddy B.V.B., Pandit M.W. (1990). Correlation Between Stability of a Protein and Its Dipeptide Composition: A Novel Approach for Predicting in Vivo Stability of a Protein from Its Primary Sequence. Protein Eng. Des. Sel..

[35] Hamdi K., Salladini E., O’Brien D.P., Brier S., Chenal A., Yacoubi I., Longhi S. (2017). Structural Disorder and Induced Folding within Two Cereal, ABA Stress and Ripening (ASR) Proteins. Sci. Rep..

[36] Pérez-Díaz J., Pérez-Díaz J.R., Medeiros D.B., Zuther E., Hong C.Y., Nunes-Nesi A., Hincha D.K., Ruiz-Lara S., Casaretto J.A. (2019). Transcriptome Analysis Reveals Potential Roles of a Barley *ASR* Gene That Confers Stress Tolerance in Transgenic Rice. J. Plant Physiol..

[37] Rogozin I.B., Carmel L., Csuros M., Koonin E.V. (2012). Origin and Evolution of Spliceosomal Introns. Biol. Direct.

[38] Kuo Y.T., Chao Y.T., Chen W.C., Shih M.C., Chang S.B. (2019). Segmental and Tandem Chromosome Duplications Led to Divergent Evolution of the *Chalcone synthase* Gene Family in *Phalaenopsis orchids*. Ann. Bot..

[39] Zhu Y., Wu N., Song W., Yin G., Qin Y., Yan Y., Hu Y. (2014). Soybean (*Glycine max*) Expansin Gene Superfamily Origins: Segmental and Tandem Duplication Events Followed by Divergent Selection among Subfamilies. BMC Plant Biol..

[40] Sadowski I., Ma J., Triezenberg S., Ptashne M. (1988). GAL4-VP16 Is an Unusually Potent Transcriptional Activator. Nature.

[41] Yu Y., Li M., Song T., Zhang S., Wang T. (2024). Genome-Wide Identification of *Miscanthus* ASR Gene Family Reveals That *MsASR4* Is Linked to NaCl Tolerance. Ind. Crops Prod..

[42] Liu H., Ding Q., Cao L., Huang Z., Wang Z., Zhang M., Jian S. (2023). Identification of the Abscisic Acid-, Stress-, and Ripening-Induced (ASR) Family Involved in the Adaptation of *Tetragonia tetragonoides* (Pall.) Kuntze to Saline–Alkaline and Drought Habitats. Int. J. Mol. Sci..

[43] Jeffares D.C., Penkett C.J., Bähler J. (2008). Rapidly Regulated Genes Are Intron Poor. Trends Genet..

[44] Xiao F., Zhou H. (2023). Plant Salt Response: Perception, Signaling, and Tolerance. Front. Plant Sci..

[45] Meena R.P., Vishwakarma H., Ghosh G., Gaikwad K., Chellapilla T.S., Singh M.P., Padaria J.C. (2020). Novel ASR Isolated from Drought Stress Responsive SSH Library in Pearl Millet Confers Multiple Abiotic Stress Tolerance in *PgASR3* Transgenic Arabidopsis. Plant Physiol. Biochem..

[46] Hu Y.-X., Yang X., Li X.-L., Yu X.-D., Li Q.-L. (2014). The *SlASR* Gene Cloned from the Extreme Halophyte *Suaeda liaotungensis* K. Enhances Abiotic Stress Tolerance in Transgenic *Arabidopsis thaliana*. Gene.

[47] Paysan-Lafosse T., Blum M., Chuguransky S., Grego T., Pinto B.L., Salazar G.A., Bileschi M.L., Bork P., Bridge A., Colwell L. (2023). InterPro in 2022. Nucleic Acids Res..

[48] Gasteiger E., Hoogland C., Gattiker A., Duvaud S., Wilkins M.R., Appel R.D., Bairoch A., Walker J.M. (2005). Protein Identification and Analysis Tools on the ExPASy Server.

[49] Ishida T., Kinoshita K. (2007). PrDOS: Prediction of Disordered Protein Regions from Amino Acid Sequence. Nucleic Acids Res..

[50] Savojardo C., Martelli P.L., Fariselli P., Profiti G., Casadio R. (2018). BUSCA: An Integrative Web Server to Predict Subcellular Localization of Proteins. Nucleic Acids Res..

[51] Madeira F., Pearce M., Tivey A.R.N., Basutkar P., Lee J., Edbali O., Madhusoodanan N., Kolesnikov A., Lopez R. (2022). Search and Sequence Analysis Tools Services from EMBL-EBI in 2022. Nucleic Acids Res..

[52] Kumar S., Stecher G., Li M., Knyaz C., Tamura K. (2018). MEGA X: Molecular Evolutionary Genetics Analysis Across Computing Platforms. Mol. Biol. Evol..

[53] Chen C., Chen H., Zhang Y., Thomas H.R., Frank M.H., He Y., Xia R. (2020). TBtools: An Integrative Toolkit Developed for Interactive Analyses of Big Biological Data. Mol. Plant.

[54] Cai K., Zeng F., Wang J., Zhang G. (2021). Identification and Characterization of HAK/KUP/KT Potassium Transporter Gene Family in Barley and Their Expression under Abiotic Stress. BMC Genom..

[55] Bailey T.L., Johnson J., Grant C.E., Noble W.S. (2015). The MEME Suite. Nucleic Acids Res..

[56] Jiang W., Tong T., Li W., Huang Z., Chen G., Zeng F., Riaz A., Amoanimaa-Dede H., Pan R., Zhang W. (2022). Molecular Evolution of Plant 14-3-3 Proteins and Function of Hv14-3-3A in Stomatal Regulation and Drought Tolerance. Plant Cell Physiol..

[57] Lescot M., Déhais P., Thijs G., Marchal K., Moreau Y., Van de Peer Y., Rouzé P., Rombauts S. (2002). PlantCARE, a Database of Plant *Cis*-Acting Regulatory Elements and a Portal to Tools for in Silico Analysis of Promoter Sequences. Nucleic Acids Res..

[58] Colmsee C., Beier S., Himmelbach A., Schmutzer T., Stein N., Scholz U., Mascher M. (2015). BARLEX—The Barley Draft Genome Explorer. Mol. Plant.

[59] Mascher M., Gundlach H., Himmelbach A., Beier S., Twardziok S.O., Wicker T., Radchuk V., Dockter C., Hedley P.E., Russell J. (2017). A Chromosome Conformation Capture Ordered Sequence of the Barley Genome. Nature.

[60] Cai K., Kuang L., Yue W., Xie S., Xia X., Zhang G., Wang J. (2022). *Calmodulin* and *Calmodulin-like* Gene Family in Barley: Identification, Characterization and Expression Analyses. Front. Plant Sci..

[61] Cai K., Song X., Yue W., Liu L., Ge F., Wang J. (2024). Identification and Functional Characterization of Abiotic Stress Tolerance-Related *PLATZ* Transcription Factor Family in Barley (*Hordeum vulgare* L.). Int. J. Mol. Sci..

[62] Livak K.J., Schmittgen T.D. (2001). Analysis of Relative Gene Expression Data Using Real-Time Quantitative PCR and the 2^-ΔΔCT^ Method. Methods.

